# Steps Toward Standardized In Vitro Assessment of Immunomodulatory Equine Mesenchymal Stromal Cells Before Clinical Application

**DOI:** 10.1089/scd.2021.0189

**Published:** 2022-01-13

**Authors:** Olivia J. Lee, Thomas G. Koch

**Affiliations:** Department of Biomedical Sciences, University of Guelph, Guelph, Canada.

**Keywords:** mesenchymal stromal cells, immunomodulation, potency, standardization, heterogeneity

## Abstract

Inflammation-associated disorders are significant causes of morbidity in horses. Equine single-donor mesenchymal stromal cells (sdMSCs) hold promise as cell-therapy candidates due to their secretory nonprogenitor functions. This has been demonstrated by mononuclear cell suppression assays (MSAs) showing that sdMSCs are blood mononuclear cell (BMC) suppressive in vitro. sdMSCs derived from umbilical cord blood are of clinical interest due to their ease of procurement, multipotency, and immunomodulatory ability. Due to the inherent donor-to-donor heterogeneity of MSCs, the development of robust and easily deployable methods of potency assessment is critical for improving MSCs' predictability in treating inflammatory diseases. This study focuses on the development of robust in vitro potency assays and the assessment of potential sdMSC therapeutic end products generated from pooled sdMSCs (pMSCs). We hypothesized that, compared to MSA using only one donor, MSA using pooled BMCs (pBMCs) is a more robust sdMSC potency assay due to reduced donor BMC heterogeneity. pBMCs were generated by pooling equine BMCs isolated from peripheral blood of five donors in equal ratios. pBMCs were labeled with carboxyfluorescein succinimidyl ester (CFSE) and stored in liquid nitrogen until use. Similarly, pooling sdMSCs from multiple equine donors in equal ratios generated pMSCs. sdMSC cultures were assessed with pBMCs in MSA using Bromodeoxyuridine ELISA and CFSE. Proliferation assessment of BMCs from individual donors revealed varied responses to concanavalin A (ConA) stimulation. MSA using BMCs from single donors further demonstrated BMC donor variability. Utilizing this assay, we have also found that the immunosuppressive potencies of pMSCs are at least equal, if not more, than the calculated mean of individual cultures. MSA based on pBMCs provides a consistent and reproducible equine sdMSC potency assay. This knowledge could be used in production monitoring of cellular potency and as release criteria before clinical use.

## Introduction

Horses are valued in sports and recreation; they also serve as preclinical models for human conditions. Inflammation-related disorders, such as osteoarthritis and inflammatory airway disease, are significant causes of morbidity in the equine industry, and these diseases affect equine species similarly to humans [1]. Currently, there is an urgent unmet clinical need for effective treatments of inflammation, as common medications, such as corticosteroids and nonsteroidal anti-inflammatory drugs (NSAIDS), are considered symptom-modifying agents with adverse side effects. Advancements in single donor mesenchymal stromal cells (sdMSCs) research offer new approaches to treating inflammation in horses and humans [2].

sdMSCs' immunosuppressive capabilities have been well documented in both humans and horses. Human sdMSCs have lymphocyte suppressive abilities in a dose-dependent manner [3]. Similarly, it has been demonstrated that equine sdMSCs are blood mononuclear cell (BMC) suppressive in vitro [4].

Furthermore, when equine sdMSCs were administered in lipopolysaccharide induced synovitis in vivo, CD4^+^ and CD8^+^ lymphocytes increased and CD4^+^/CD8^+^ double-positive lymphocytes decreased [5]. The increase in CD4^+^ and CD8^+^ lymphocytes may be attributed to the increase in CD4^+^ helper T cells and CD8^+^ cytotoxic T cell populations. The presence of CD4^+^/CD8^+^ double-positive T cells is associated with several autoimmune conditions, such as autoimmune thyroiditis, systemic sclerosis, and in the fluid of joints from rheumatoid arthritis patients. Furthermore, higher CD4^+^/CD8^+^ double-positive T cell numbers have been shown to be associated with Kawasaki disease [6].

One of the main challenges for the clinical implementation of sdMSC therapeutics is the development of robust, consistent, and easily deployable end products [7]. Efforts to minimize donor–donor and batch–batch variability are important in strengthening the manufacturing process of sdMSC therapeutic products, as predictable, standardized, and effective sdMSC products may lead to more consistent therapeutic outcomes [8,9].

sdMSCs are a heterogeneous population of cells with differential functional capabilities that depend on the health status of the donor, tissue of origin, isolation methods, and culture expansion methodologies [8,10]. Thus, it is crucial to confirm that the sdMSCs exert the intended effect through potency assays. Through measuring the biological activity of the sdMSC products, the comparability between different lots of the product can be verified [9]. The potency test would also serve as the basis to compare different types of sdMSC products [11]. Furthermore, if changes are introduced to the manufacturing process, a robust potency assay with defined acceptance criteria will ensure manufacturing consistency and that the recipient would be treated with a consistent and potent cellular therapy product [12].

The mononuclear cell suppression assay (MSA) can be used for evaluating sdMSCs' immunosuppressive potency in vitro. The most common MSA uses responder BMCs from one donor and either allogeneic stimulator cells, mitogens such as concanvalin A (ConA), or CD3/CD28 beads as stimulation for BMC proliferation [13]. The addition of sdMSCs to this system suppresses T lymphocyte proliferation. By measuring the extent of suppression, sdMSCs' immunosuppressive potency can be quantified.

For this study, we hypothesized that, compared to MSA using only one donor, MSA using pooled BMCs (pBMCs) is a more robust sdMSC potency assay due to reduced donor BMC heterogeneity. Furthermore, the pooling of sdMSCs from multiple donors allows the generation of standardized effective MSC products. In this study, we used a robust potency assay using pBMC and explored the immunosuppressive potency of pooled sdMSCs (pMSCs).

## Materials and Methods

### Ethics statement

The University of Guelph Animal Care Committee specifically approved this study with regards to the procedures of equine peripheral blood lymphocytes (animal use protocol 1756) and equine umbilical cord blood (animal use protocol 1570). Additional research conducted using specimens of this kind does not require review by the Animal Care Committee (falls under CCAC Category of Invasiveness A). Peripheral blood and cord blood collection were conducted as add-on procedures to the routine care of the horse. No animals were harmed or sacrificed during this study.

The horse owners/agents provided informed consent in writing. Cord blood was collected after the umbilical cord had been clamped and detached from the foal. Access to research horses is granted after the approval of the animal care protocol by investigators. Peripheral blood was collected from adult horses at the Equine Research Station managed by the University of Guelph in partnership with the Ontario Ministry of Agriculture, Forestry, and Rural Affairs. Peripheral blood was collected by Dr. Koch. Horses were under mild sedation (Xylazine HCl, 0.35–0.40 mg/kg bwt IV; Bayer, Toronto, ON, Canada), and blood was collected from the jugular vein following which manual pressure was applied for several minutes to aid hemostasis.

### sdMSC isolation and culturing

Umbilical cord blood-derived sdMSCs were isolated from newborn foals as previously described [14]. Briefly, 1XRBC lysis buffer (1.5 M NH_4_CL, 100 mM KHCO_4_, 1 mM EDTA, pH 7.3; Sigma, Oakville, Canada) was added to cord blood in 3:1 ratio and mixed for 10 min at room temperature. Then the mixture was centrifuged for 10 min at 400 *g*. The supernatant was discarded, and the cell pellet was washed in PBS (Sigma) and centrifuged. The supernatant was discarded, and cells were resuspended in sdMSC isolation media consisting of DMEM low glucose (Lonza, Walkersville, MD), 10% fetal bovine serum (FBS) (Invitrogen, Burlington, Canada), 100 nM dexamethasone (Sigma), 2 mM l-glutamine (Sigma), and 100 U penicillin–streptomycin (Invitrogen) and seeded in culture flasks at density 1 × 10^6^ cells cm^−2^, incubated at 38°C and 5% CO_2_.

Media were changed every 24 h for the first 3 days in culture and every 3 days thereafter. After the formation of putative sdMSC colonies, the adherent cells were detached with 0.25% trypsin (Sigma) and plated again in sdMSC expansion media consisting of DMEM low glucose (Lonza), 10% FBS (Invitrogen), 2 mM l-glutamine (Sigma), and 100 U penicillin–streptomycin (Invitrogen). Cells were passaged at 70% confluency and seeded at 5000 cells/cm^2^.

### Isolation of BMCs from peripheral blood

Peripheral blood was obtained from the jugular vein of adult horses. BMCs were isolated using Ficoll-density gradient. Fifteen milliliters of Ficoll-Paque Plus (density 1.078 g/mL; STEMCELL Technologies, Vancouver, Canada) was loaded with 35 mL of undiluted whole blood. Gradients were centrifuged at 300 *g* for 30 min with no break. The BMC containing fraction was removed, pooled, and washed with PBS. The remaining pellet after supernatant removal was washed again with PBS and resuspended in Roswell park memorial institute (RPMI) 1640 medium supplemented with penicillin (100 IU/mL; Invitrogen), streptomycin (0.1 mg/mL; Invitrogen), and 10% FBS (Invitrogen), and a live cell count was performed with the NC-100 automatic cell counter (Chemometec, Denmark).

For carboxyfluorescein succinimidyl ester (CFSE) labeled BMCs, the cells were labeled with CellTrace™ CFSE cell proliferation Kit (ThermoFisher) following the manufacturer's protocol. BMCs were resuspended in CryoStor^®^ (STEMCELL technologies) at a concentration of 6 × 10^6^ cells/mL. BMCs were frozen and stored in liquid nitrogen until use.

### Flow cytometric characterization of sdMSCs and BMCs

Cord-blood-sdMSCs were chemically detached with Accumax (STEMCELL Technologies) and washed with flow buffer (PBS, 5 mM EDTA, 1% horse serum, and 0.1% sodium azide). sdMSCs were characterized with flow cytometry for antigens: CD4 (clone: CVS4; abdSerotec, Raleigh, NC), CD8 (clone: HTI4A; abdSerotec), CD11a/18 (clone: 116.2D11B10; abdSerotec), CD24 (clone: 4B4- Fluorescein isothiocyanate (FITC), Beckman Coulter, Mississauga, Canada**)**, CD44 (clone: CVS18, abdSerotec), CD45 (clone: DH16A; VMRD), CD73 (clone: 10f1; Abcam, Toronto, Canada), CD90 (clone: DH24A; VMRD, Pullman, WA), MHC I (clone: 117.1B12C11; abdSerotec), and MHC II (clone: 130.8E8C4; abdSerotec).

Cells were incubated at 4°C in the dark for 15 min, followed by a wash and secondary antibody incubation at 4°C for 15 min in the dark, rat anti-mouse IgM-FITC or goat anti-mouse IgG1-FITC (Abcam) for CD90 were used as secondary antibodies. Ammonium chloride (Sigma-Aldrich) red blood cell lysis was performed on peripheral and cord blood for leukocyte isolation followed by a wash with flow buffer. sdMSCs were stained for CD4, CD8, CD11a/18, CD45, CD90, MHC I, and MHC II surface expression. A minimum of 10,000 events was acquired for each antibody with CellQuest software (Becton Dickinson), and data were analyzed with FlowJo software (Tree Star, Inc., Ashland, OR). Gates to identify leukocytes or sdMSC populations were consistent throughout all experiments.

### MSA and pooled MSA

**FIG. 1.**
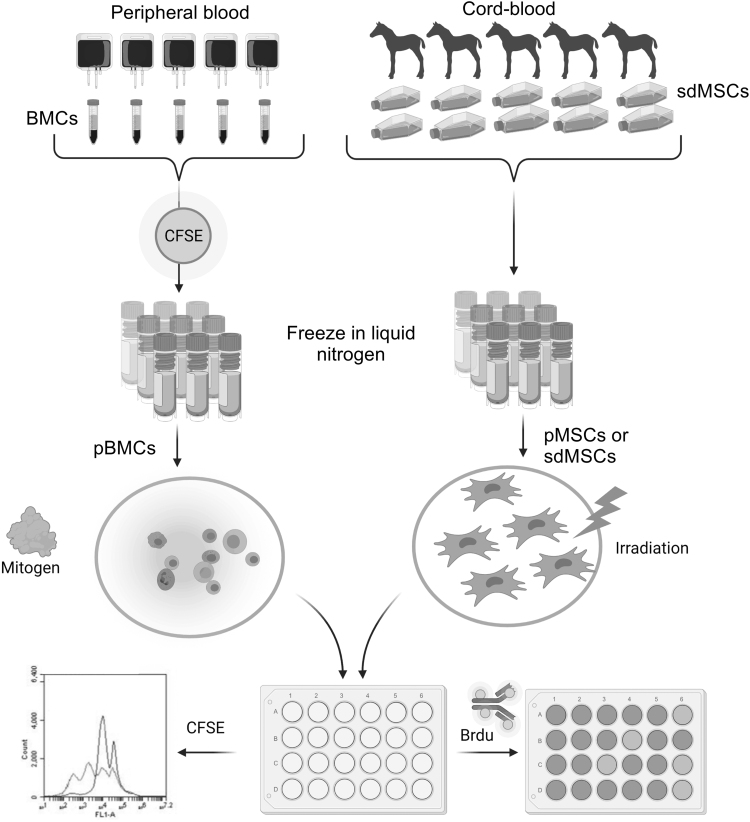
Methodology of pooling of sdMSCs (pMSCs) and labeling of pBMCs with CFSE. Equine BMCs were isolated from peripheral blood using Ficoll-density gradient. BMCs were pooled in equal ratios from five donors. The pBMCs to be used in CFSE experiments were labeled with CellTrace™ CFSE Cell Proliferation Kit (ThermoFisher) following the manufacturer's protocol. pBMCs were frozen and stored in liquid nitrogen until use. Umbilical cord blood derived sdMSCs were isolated from newborn foals, characterized, and frozen until use. sdMSCs from passage 2–5 were cocultured with pBMCs at a ratio of 1:10 sdMSC as determined by previous optimization experiments. pBMCs were stimulated with 5 μg/mL of ConA. After 3 days of culture, BrdU was added and cocultures were incubated for an additional 18 h. Then proliferation was measured by BrdU ELISA or CFSE. CFSE, carboxyfluorescein succinimidyl ester; sdMSC, single-donor mesenchymal stromal cell; pBMCs, pooled blood mononuclear cells; BrdU, bromodeoxyuridine; ConA, concanavalin A.

MSA and pooled MSA (pMSA) were used to evaluate sdMSCs for their ability to suppress BMC proliferation in vitro (Fig. 1). Positive control, consisted of either BMCs from a single donor in MSA or pBMCs from five donors mixed in equal ratios in pMSA, were stimulated with 5 μg/mL of ConA (Sigma). Negative control cultures of BMC or pBMCs were not stimulated with mitogen. sdMSCs at passages 2–5 were irradiated (20 Gy) and cocultured with ConA stimulated BMCs or pBMCs at a ratio of 1:10.

The culture media for all groups in MSA and pMSA consisted of RPMI 1640 basal media supplemented with penicillin (100 IU/mL; Invitrogen), streptomycin (0.1 mg/mL; Invitrogen), and 10% FBS (Invitrogen). Cocultures were performed in 48-well plates, and reactions were incubated for 4 days at 38°C in 5% CO_2_. After 3 days of coculturing, cells were stained with Bromodeoxyuridine (BrdU) for 18 h and assessed with a BrdU ELISA Kit (Roche, Mississauga, Canada) following the manufacturer's protocol. Fold changes of BMC proliferation are calculated using the formula below:

$$Foldchangesrelativetothenegativecontrol=\frac{Absorbance\left(370nm\right)ofsdMSC:BMCco-culture}{AverageAbsorbance\left(370nm\right)ofeachindividualBMCdonor}$$

For CFSE pMSA, sdMSCs were collected and washed once with PBS and twice with flow buffer (1 × PBS, 5 mM EDTA [Promega, Madison, WI], 1% horse serum [ThermoFisher], 0.1% sodium azide [Sigma]). Subsequently, 7-aminoactinomycin D (Sigma) was added for 5 min. Samples in triplicates were run and analyzed on a BD Accuri C6 cytometer unit (BD Biosciences, San Jose, CA).

### Statistical analysis

Results were analyzed using GraphPad Prism Software Version 8.0 (GraphPad Software, San Diego, CA). Data are presented as mean ± standard error of the mean (SEM). MSA and pMSC data were analyzed using one-way ANOVA and corrected using Tukey's post-hoc test, at *P* < 0.05 (* indicates *P* < 0.05).

## Results

### BMC donor-to-donor heterogeneity exists and affects sdMSC potency readouts

**FIG. 2.**
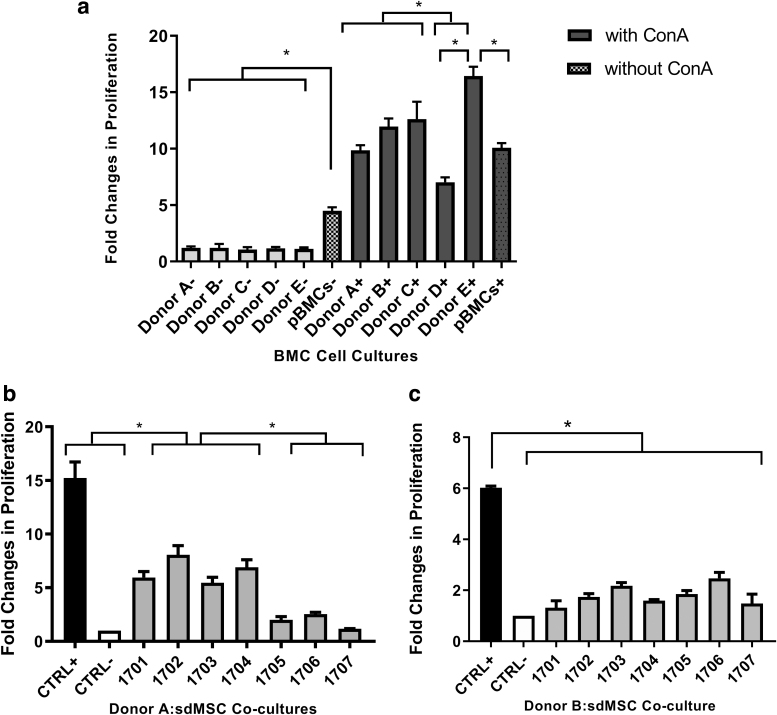
Donor-to-donor variability of peripheral BMCs. **(a)** Proliferation of BMCs from five donors in absence (−) and presence (+) of 5 μg/mL of ConA. The pBMCs were generated by pooling BMCs in equal proportions. BMCs were plated onto 48-well culture plates immediately after thawing. sdMSCs were irradiated with 20 Gy before coculturing with pBMCs. BrdU was added to the cell cultures after 3 days, and cells were incubated for an additional 18 h. BMC proliferation was measured with BrdU ELISA. **(b, c)** BMC suppression assay of seven sdMSC cultures assessed with BMCs from two donors: donor A **(b)** and donor B **(c)**. The BMC suppression was measured by BrdU ELISA. *P < 0.05 and signifies significant differences between cell cultures and/or controls.

To determine whether donor-to-donor variability exists between BMC donors, we quantified the proliferation of BMCs isolated from five donors after stimulation with ConA. Our results show that the extent of BMC proliferation differs significantly between BMC donors, demonstrating that BMCs' response to stimuli is donor dependent (Fig. 2A). To determine whether BMCs from different donors would respond to sdMSCs differently, sdMSCs (*n* = 7) were cocultured with BMCs from two donors. As expected, the positive controls, where BMCs were stimulated with ConA and cultured without sdMSCs, proliferated to different extents for each donor: by a fold change of 15 for donor A and by a fold change of 6 for donor B.

All seven sdMSC cultures were able to suppress BMCs relative to the positive control. sdMSC cultures 1705, 1706, and 1707 were more immunosuppressive than 1701, 1702, 1703, and 1704 when tested against donor A. However, all seven sdMSC cultures showed similar immunosuppressive potencies when tested against donor B (Fig. 2B, C). The difference in BMC suppression patterns was observed using identical sdMSC cultures, but different BMC donors indicated that BMC donor-to-donor variability exists. Therefore, the MSC potency test using just one BMC donor may be biased.

### pMSA can be used to minimize BMC donor-to-donor variability

BMCs were pooled from five donors in equal ratios to minimize BMC donor-to-donor variability. Then we tested whether sdMSCs were able to suppress pBMCs as they did when cocultured with single donor BMCs. ConA was added to all cocultures except for the negative control; thus, in addition to mixed lymphocyte stimulation between BMC cultures, mitogen stimulation was also present.

**FIG. 3.**
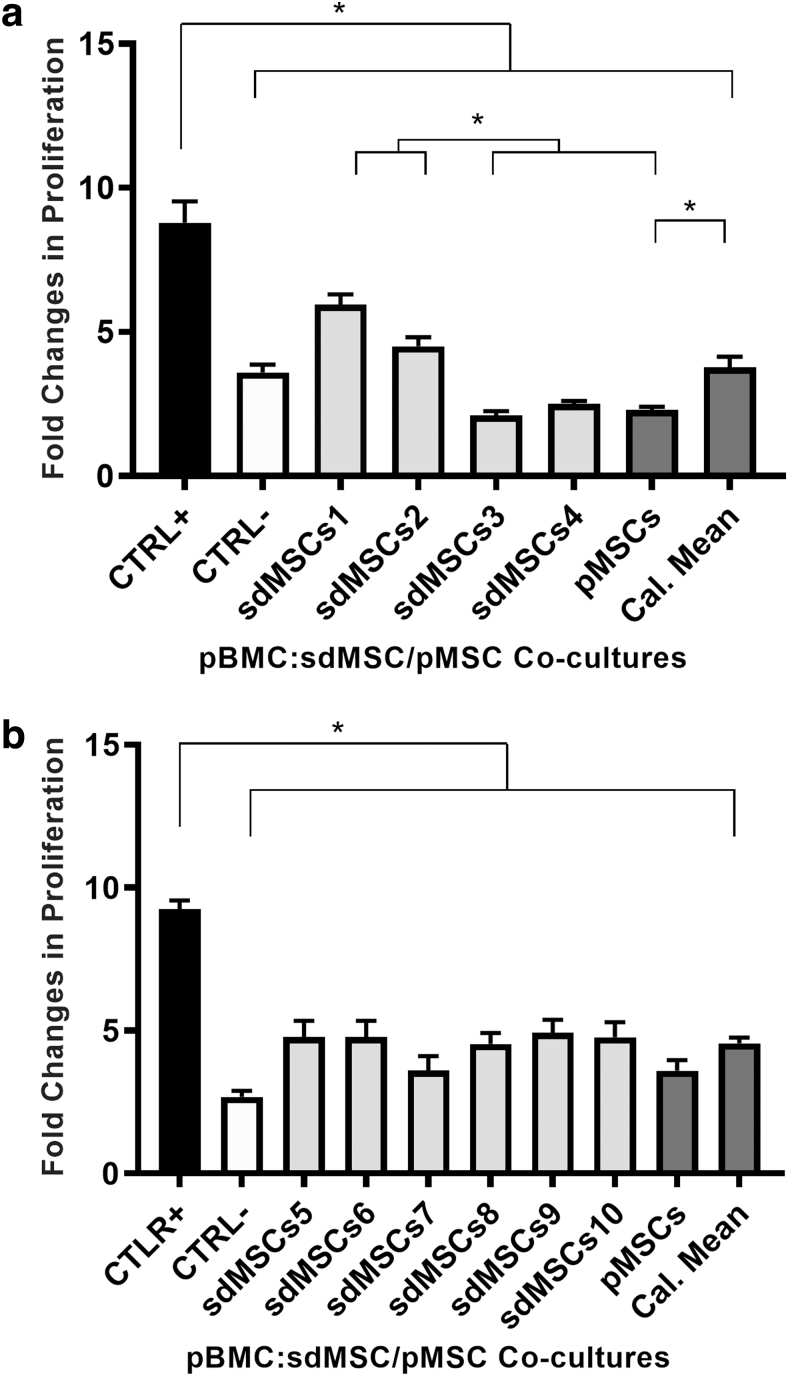
pMSA of four **(a)** and six **(b)** sdMSC cultures. pBMCs were generated from five donors mixed in equal ratios (*n* = 5). pMSCs were generated by pooling sdMSCs used in each assay in equal ratios. The pMSCs were irradiated with 20 Gy before coculturing with pBMCs. Five μg/mL of ConA was added to all cultures except for the negative control (CTRL-). The pBMC suppression was measured by BrdU ELISA. BrdU was added to the pBMC:MSC cocultures after 3 days, and cells were assessed after 18 h of incubation. The calculated mean was determined by averaging all values obtained for each sdMSC cultures. *P < 0.05. pMSA, pooled mononuclear cell suppression assay.

**FIG. 4.**
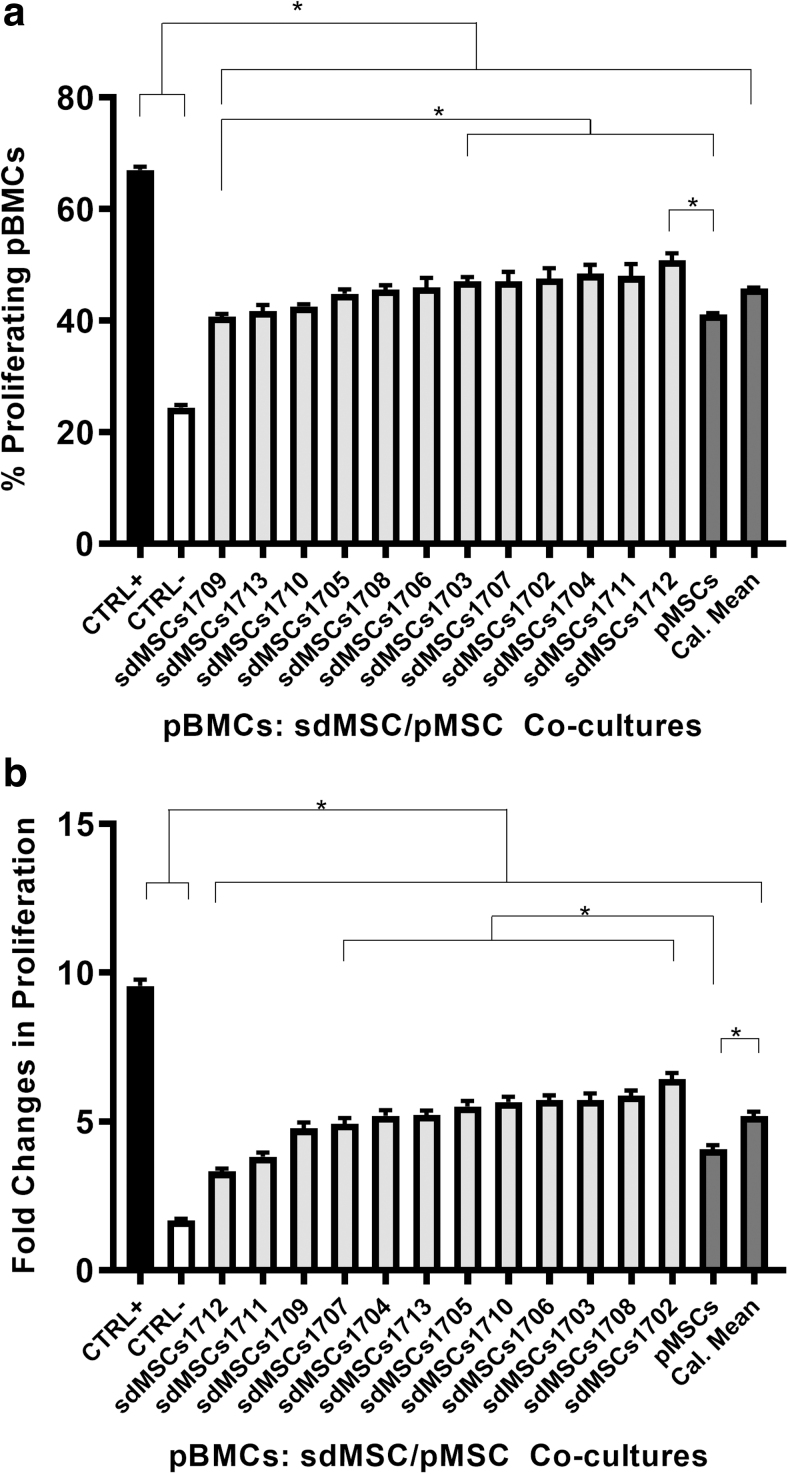
pMSA using 12 sdMSC cultures. The pMSC culture consists of the 12 sdMSC cultures mixed in equal ratios. The pMSCs were irradiated with 20 Gy before coculturing with pBMCs in 48 well plates. pBMCs were generated from five donors (*n* = 5). For pBMCs used in CFSE experiments, the cells were labeled with CFSE immediately after isolation and stored in liquid nitrogen until use. ConA was added at a concentration of 5 μg/mL to all cocultures except for the negative control (CTRL-). The calculated mean (Cal. Mean) was determined by averaging all values obtained for each sdMSC cultures. pBMC proliferation was assessed with CFSE **(a)** and BrdU **(b)**. *P < 0.05.

As a preliminary test, four sdMSC cultures were added to pBMCs, and proliferation was assessed with BrdU ELISA. All four sdMSC cultures were able to suppress pBMC proliferation (Fig. 3A). We repeated the test with six sdMSC cultures and had similar results (Fig. 3B). To further increase the power of the test, we then tested 12 sdMSC cultures against pBMCs (Fig. 4) using BrdU ELISA and CFSE. Again, all 12 sdMSC cultures suppressed pBMC proliferation. The suppressive potency between cultures was statistically different, as some sdMSC cultures suppressed pBMC proliferation more than others. Furthermore, the most immunosuppressive cultures measured by CFSE differ from those measured by BrdU ELISA.

### pMSCs suppress pBMC activity

To determine if pMSCs also suppress pBMCs, sdMSC cultures used in each of the experiments were pooled in equal ratios and their immunosuppressive potencies were assessed. It should be noted that sdMSCs were individually expanded and harvested before being pooled in equal numbers, as opposed to being pooled and then culture expanded before inclusion in the MSA as reported by others [15]. This approach was selected to avoid the risks of unequal representation of each sdMSC donor due to different proliferation rates during expansion.

pMSCs were able to suppress pBMCs in all three experiments. Interestingly, pMSCs suppressed pBMC proliferation more than the calculated mean of individual cultures in the experiments using four sdMSC cultures (Fig. 3A) and 12 sdMSC cultures (Fig. 4B), suggesting that pMSCs not only are capable of serving as alternatives to individual sdMSC cultures but also that they may be more effective in suppressive pBMC proliferation.

## Discussion

Our data demonstrated that BMC donor-to-donor heterogeneity does exist; therefore, evaluating sdMSC potency using only one BMC donor could confound potency readouts. In this study, we have developed an in vitro potency assay using equine pBMCs that minimize biases that result from BMC donor-to-donor variations. Furthermore, we used this assay to evaluate the potency of pMSCs and demonstrated that they are equally or more immunosuppressive than individual sdMSC cultures. pMSC may, therefore, represent a therapeutic product with improved consistency and decreased production time.

Our observation that substantial donor-to-donor variability was present in the unfractionated BMCs collected from individual donors agrees with several previous studies [16,17]. This variability could be explained by ConA's mode of action. ConA only stimulates T cell proliferation; however, the percentage of T cells can vary significantly, from 45% to 70%, depending on the health condition of the donor, thus leading to differential responses to the stimulation.

The concept of using pBMCs to minimize variability has been documented by other immunological assays [16–19]. Unlike previous studies, this study captured both allo-response between BMCs and mitogenesis by having ConA and pBMCs in a unique system. The results of the pMSA tests using 4, 6, and 12 sdMSC donors were consistent and comparable, demonstrating the reliability and repeatability of the pMSA assay.

Surprisingly, even though both BrdU ELISA and CFSE allowed the reliable quantification of pBMC proliferation, these techniques yielded different results for the relative suppressive potency between sdMSC cultures. This discrepancy could be attributed to the different time frames within the coculture duration that each proliferation test captures. BrdU was added on day 3 of coculture, and pBMC proliferation was only measured between day 3 and 4. In contrast, pBMCs were prelabeled with CFSE before coculturing, and proliferation during the entire process was captured by CFSE. Thus, it is possible that the sdMSC cultures with the highest pBMC suppressive potency on day 3 may not be the same as those with the highest potency throughout the entire coculture period. This suggests that labeling with CFSE may provide a broader view of sdMSCs' immunosuppressive potency.

CFSE is superior to BrdU ELISA in terms of deployability and flexibility, providing additional advantages. The CFSE method allows prelabeling and cryopreserving of pBMCs until use, which can be tested off-the-shelf for allo-stimulated, mitogen-driven proliferation assay. If further analysis of BMC subpopulations is required, CFSE provides the flexibility of simultaneous labeling with multiple markers.

The results demonstrating that pMSCs are capable of suppressing pBMC proliferation agreed with Ketterl and colleagues (2015) with one exception: in Figs. 3A and 4B, the pMSCs had greater immunosuppressive potencies than the calculated mean of individual donors, whereas, in their study, pMSCs reduced T cell proliferation to the same extent as the arithmetic means of individual donors. This discrepancy may stem from the methodology used for assessing pBMC proliferation; the results in Figs. 3A and 4B were obtained by BrdU, whereas results from Ketterl et al. were obtained using CFSE. Our observations are further buttressed by in vivo studies by other groups. Administration of bone marrow-derived, cultured, pooled, allogeneic sdMSCs had demonstrated efficacy in the treatment of critical limb ischemia [20].

In addition, Kuçi et al. (2016) demonstrated that patients treated with clinical end products generated by pooled bone marrow mononuclear cell (BM-BMC) derived sdMSCs had a higher overall survival rate than patients treated with sdMSCs from single donors. Intriguingly, their study also showed that sdMSCs derived from pooled bone marrow mononuclear cells suppressed BMC proliferation more than sdMSC cultures pooled upon coculturing [15]. The pooled BM-BMC method differs from our pMSA as pooled BM-BMCs were grown in culture for 14 days to isolate pMSCs and then further cultured until passage two. The long culture duration before coculturing may lead to unequal proportions of sdMSCs from each donor—the most dominant sdMSCs may outcompete other sdMSC cultures. In our experiment, pMSCs were pooled upon coculturing to ensure equal ratios from each sdMSC donor.

pMSCs have several advantages over sdMSCs. First, multiple studies have shown that sdMSCs exhibit donor-specific variability [21–23]; pooling of sdMSCs from multiple donors makes therapeutic products more predictable [16,24]. In addition, pMSCs allow the generation of sdMSC banks—large quantities of the same sdMSC batch that contains sufficient numbers of sdMSCs for several treatments, which allows greater standardization between treatments. It is possible that through pooling sdMSCs, one may not achieve the best outcome possible using the most potent sdMSCs, but simultaneously, one may avoid, or at least minimize, the risk of not having any effects at all.

Second, due to the high cell number required for sdMSC therapy, culture extensive expansion is required; however, previous studies have reported that long-term in vitro culture expansion increases sdMSCs' genetic instability [25–27]. Importantly, long-term culture expansion reduced the immunomodulatory capacity of sdMSCs. pMSCs allow large amounts of sdMSCs to be generated without extensive culture expansion; thus, producing higher quality, safer, and more consistent therapeutic products. Less culture expansion simultaneously implies a lower requirement for production time. pMSCs could serve as an immediate solution for the mitigation of adverse acute inflammatory responses.

One limitation of this study is that the assessment of pMNC proliferation does not provide mechanistic insights to sdMSCs' immunosuppression; information such as the cytokines involved or suppression of subpopulations cannot be determined through quantifying the proliferation alone. Thus, we propose that future studies on elucidating the cytokine changes induced by pMSA will be necessary. In addition to offering mechanistic insights, cytokine analysis also increases the sensitivity of the assay, as small changes that are not detectable by measuring proliferation can be detected by measuring changes in cytokine levels.

Even though in vitro pMSA offers faster and more economical solutions [7], preclinical studies are essential for moving forward to clinical investigations. Thus, future investigations aimed at linking in vitro data with in vivo efficacy would be needed. Previous studies have suggested that in vitro data may have predictive values in preclinical models. Deskins et al. (2013) showed that sdMSC cultures with the highest performance in growth rate, proliferation, and cell viability in vitro also had the highest engraftment and vascularity capacity in vivo. In other studies, pretreatment of sdMSCs led to higher MNC suppression in vitro and enhanced therapeutic activity in animal models in vivo [28–30].

Thus, future research avenues could consider administering the sdMSC cultures with the poorest and the best performance assessed by pMSA in preclinical models and utilize changes in cytokine levels and gene expression to elucidate the association between in vitro sdMSC potency determined with pMSA and in vivo efficacy. If a predictive panel of cytokines can be determined for the most potent sdMSC culture or pMSC batch, then it will be possible to create a faster and more sensitive in vitro potency test.

## Conclusion

Our results demonstrate that equine BMC donor-to-donor heterogeneity exists; thus, in vitro sdMSC potency tests based on one donor may be difficult to interpret reliably. pMSA based on pBMCs offers a more standardized potency assessment solution through minimizing BMC donor-to-donor heterogeneity. Importantly, using pMSA, we have shown that pMSCs have immunosuppressive potential. Compared to sdMSCs from single donors, pMSCs are superior in minimizing sdMSC donor-to-donor variability and are more predictable because it allows the generation of sdMSC banks which can be used for multiple treatment. Furthermore, pMSCs also require less culture expansion. If future studies could demonstrate that pMSCs have strong potency in vivo, then pMSCs can become promising therapeutic products for treating inflammatory diseases.
